# Finite-time synchronization of different dimensional chaotic systems with uncertain parameters and external disturbances

**DOI:** 10.1038/s41598-022-19659-7

**Published:** 2022-09-14

**Authors:** Juan Li, Jiming Zheng

**Affiliations:** 1 School of Science, Chongqing University of Posts and Telecommunications, Chongqing, 400065 China; 2 Key Laboratory of Intelligent Analysis and Decision on Complex Systems, Chongqing University of Posts and Telecommunications, Chongqing, 400065 China

**Keywords:** Engineering, Mathematics and computing

## Abstract

This paper proposes a new control scheme using two scaling matrices that realizes the finite-time synchronization of different-dimensional chaotic systems with parameter uncertainties and external disturbances. Firstly, based on Lyapunov stability theorem and finite-time stability theorem, the definition of finite-time synchronization of chaotic systems with different dimensions is introduced. Secondly, in the case of external disturbance and parameter uncertainty, an adaptive feedback hybrid controller and parameter adaptive laws are designed to synchronize different dimensional uncertain chaotic systems in finite-time. Then, according to the characteristics of the unknown parameters of the system, a transformation matrix is constructed to meet the needs of chaotic systems with different dimensions, and a simplified synchronization control scheme is designed. Finally, two numerical experiments are carried out to verify the effectiveness of the proposed methods.

## Introduction

Chaotic systems are sensitive to initial values, and small changes in initial conditions will lead to significant differences in the final dynamic behavior of chaotic systems^1^. Since Pecora and Carroll put forward the principle of chaos synchronization for the first time in 1990^2^, which has aroused the interest of many scholars and began to be widely researched, especially in secure communication and statistical prediction^3–6^.

Finite-time synchronization is the application of the finite-time stability theorem to the synchronization research of chaotic systems^7,8^, according to the obtained error dynamic systems, to design a suitable controller so that the error system converges to zero in a finite time. Based on the finite-time stability theorem, a class of nonlinear feedback controllers is proposed in^9^, which realizes the finite-time function projection synchronization in complex networks with time delay, and the criterion of convergence time is given. Moreover, the finite-time synchronization of a class of hyperchaotic systems is researched based on the principle of feedback passivation control^10^.

However, in the actual design process of the synchronization scheme, some uncertain factors of a chaotic system may impact the synchronization of the system, and how to reduce the impact of these factors is particularly important. A nonlinear adaptive control synchronization method is designed for two identical Lorenz systems with unknown parameters^11^. The finite-time synchronization of the integer and fractional order of a homogeneous chaotic system with random perturbations and unknown parameters is researched by using adaptive control methods and Lyapunov stability theorem in^12,13^. These results show that the finite-time theorem is valuable in the synchronization control of uncertain chaotic systems and practical engineering.

With the discovery of synchronization between cardiopulmonary systems with different dimensions in the biological world^14^, the synchronization of chaotic or hyperchaotic systems with different dimensions began to attract people's interest^15–17^. Some theoretical results for the synchronization between different dimensional chaotic systems have been obtained^16,18–21^. The generalized synchronization of chaotic systems with different dimensions is researched based on the finite-time stability theorem in^18^. By reducing the size of a high-dimensional chaotic system, an adaptive controller is designed in^16^ to realize the synchronization of two different dimensions of chaotic systems. The generalized finite-time synchronization of different dimensional chaotic systems with uncertain parameters studied using the finite-time stability theorem is researched in^19,20^. Taking external disturbances and unknown parameters as the overall uncertainty factors, the finite-time synchronization of uncertain chaotic or hyperchaotic systems with different dimensions is researched in^21^ by increasing or reducing dimensions.

Many researchers have done much research on the finite-time synchronization control of two identical or unidentical chaotic systems, and the above literature gives us great research motivation.

1. Based on the existing literature, according to the characteristics of the chaotic system, we try to design a new control scheme with a faster convergence speed.
2. External disturbance exists objectively. In many literatures, the disturbance is considered a constant value and is directly canceled out in the controller. This paper tries to control the disturbance as a bounded parameter.
3. The complex behavior of the chaotic system is closely related to its dimension. Synchronizing chaotic systems of different dimensions in a limited time puts forward higher controller design requirements and brings significant challenges to our work.
4. In communication, the design of different dimensions of the driving system and response system can increase the confidentiality of information. Therefore, the synchronization of chaotic systems with different dimensions began to attract interest in communication encryption, which dramatically motivates our work.

Therefore, based on the above kinds of literature, this paper further researches the finite-time synchronization of chaotic systems with the above factors. The main work of this paper is to investigate the application of scaling matrices finite-time synchronization in different dimensional chaotic or hyperchaotic systems with external disturbances and uncertain parameters. For a class of chaotic systems with different dimensions, in the case of external disturbance and parameter uncertainty, based on the Lyapunov stability theorem and finite-time stability theorem, the corresponding controller and parameter adaptive laws are designed by using scaling matrices so that the chaotic systems with different dimensions can achieve synchronization under the same dimension infinite time. And the expression of the time when the system reaches stability is given.

This paper is arranged as follows: In section “Preliminaries”, the finite-time synchronization theory of different dimensional chaotic systems is introduced in detail. In section “Main results”, the synchronization schemes of different dimensional chaotic systems with external disturbances and uncertain parameters are presented. In section “Numerical simulations”, we will choose two groups of examples to verify the validity of the proposed methods. Finally, the conclusions and some prospects are given in section “Conclusions”.

## Preliminaries

Consider the drive system that can be described by

1 $$\dot{\user2{x}} = f\left( {\varvec{x}} \right){\varvec{\alpha}} + F\left( {\varvec{x}} \right) + {\varvec{h}}\left( t \right)$$

where ${\varvec{x}} \in {\varvec{R}}^{n}$ is the state variable, ${\varvec{\alpha}}{ = }\left( {\alpha_{1} ,\alpha_{2} , \ldots ,\alpha_{{\tau_{1} }} } \right)^{T}$ are parameters, $\tau_{1}$ is a constant, $f\left( {\varvec{x}} \right):{\varvec{R}}^{n} \to {\varvec{R}}^{{n \times \tau_{1} }}$ is the function matrix,$F\left( {\varvec{x}} \right):{\varvec{R}}^{n} \to {\varvec{R}}^{n}$ is the nonlinear function vector, ${\varvec{h}}(t) = (h_{1} \left( t \right), \ldots ,h_{n} \left( t \right))^{T} \in {\varvec{R}}^{n \times 1}$ is the external disturbance function vector.

The response system is given as

2 $$\dot{\user2{y}}{\text{ = g}}\left( {\varvec{y}} \right){\varvec{\beta}} + G\left( {\varvec{y}} \right) + {\varvec{H}}\left( t \right) + {\varvec{u}}$$

where ${\varvec{y}} \in {\varvec{R}}^{m}$ is the state variable, ${\varvec{\beta}}{ = }\left( {\beta_{1} ,\beta_{2} , \ldots ,\beta_{{\tau_{2} }} } \right)^{T}$ are parameters,$\tau_{2}$ is a constant, $g\left( y \right):{\varvec{R}}^{m} \to {\varvec{R}}^{{m \times \tau_{2} }}$ is the function matrix,$G\left( {\varvec{y}} \right):{\varvec{R}}^{m} \to {\varvec{R}}^{m}$ is the nonlinear function vector, ${\varvec{H}}(t) = (H_{1} \left( t \right), \ldots ,H_{m} \left( t \right))^{T} \in {\varvec{R}}^{m \times 1}$ is the external disturbance function vector, ${\varvec{u}} = \left( {u_{1} , \ldots ,u_{m} } \right)^{T} \in {\varvec{R}}^{m}$ is the controller to be designed.

### Definition 1

For the chaotic or hyperchaotic systems (1) and (2), if there exists a controller ${\varvec{u}} \in {\varvec{R}}^{m}$ and given matrices $\Theta \in {\varvec{R}}^{d \times m}$ and $\Phi \in {\varvec{R}}^{d \times n}$ such that the synchronization error ${\varvec{e}} =\Theta {\varvec{y}} -\Phi {\varvec{x}}$, satisfies that

3 $$\begin{gathered} \mathop {\lim }\limits_{t \to T} \left\| {{\varvec{e}}\left( t \right)} \right\| = 0 \hfill \\ \left\| {{\varvec{e}}\left( t \right)} \right\| \equiv 0,t > T \hfill \\ \end{gathered}$$

where $d$ is a certain constant, $\left\| \bullet \right\|$ is the vector norm. Then, the drive system (1) and the response system (2) are said to be finite-time synchronization under dimension $d$, where $T$ is the synchronization time of the two chaotic systems.

### Remark 1.

For our work, we give the following definitions in advance.

1. For matrix $${\rm A} \in {\varvec{R}}^{m \times n}$$:
  ${\rm A}\left( i \right)\left( {i = 1,2, \ldots ,m} \right)$ is defined as the vector composed of the elements of the $i$-th row of a matrix $\rm {A}$.
  ${\rm A}\left( {ij} \right)\left( {i = 1,2, \ldots ,m,j = 1,2, \ldots ,n} \right)$ is defined as the element of the $i$-th row and $j$-th column of a matrix $\rm {A}$.
2. Define vector function $S\left( \kappa \right) = \left( {sign\left( {\kappa_{1} } \right), \ldots ,sign\left( {\kappa_{m} } \right)} \right)^{T}$, $\left| \kappa \right|^{\lambda } = \left( {\left| {\kappa_{1} } \right|^{\lambda } , \ldots ,\left| {\kappa_{m} } \right|^{\lambda } } \right)^{T}$, $\lambda$ is any real number, where $\kappa = \left( {\kappa_{1} , \ldots ,\kappa_{m} } \right)^{T}$.
3. Define vector matrix $D\left( \kappa \right) = diag\left( {\kappa_{1} , \ldots ,\kappa_{m} } \right)$, where $\kappa = \left( {\kappa_{1} , \ldots ,\kappa_{m} } \right)^{T}$.

### Assumption 1.

The constant $d$ satisfies $0 < d \le \max \left\{ {m,n} \right\}$. We choose $d = m$ in this paper for convenience.

### Assumption 2.

The matrix $\Theta$ is row full rank matrix. Defined $\Theta^{{{ - }1}}$ as the right inverse of a matrix $\Theta$, i.e.$\Theta \Theta^{{{ - }1}} = E$.

### Assumption 3.

The external disturbance functions $h_{i} \left( t \right)(i = 1,2, \ldots ,n)$ and $H_{j} \left( t \right)(j = 1,2, \ldots ,m)$ are bounded.

### Proposition 1

^8^Consider the following system

4 $$\dot{z}\left( t \right) = \xi \left( {z\left( t \right)} \right),\xi \left( 0 \right) = 0,z\left( 0 \right) = z_{0} ,z \in D$$

where $D \subseteq {\varvec{R}}^{n}$ and $\xi :D \to {\varvec{R}}^{n}$ is continuous. Suppose there exist a Lyapunov function $V:D \to {\varvec{R}}$, positive.

real numbers $p,q$ and $\mu \in \left( {0,1} \right)$, and an open neighborhood $U \subseteq D$ of the origin such that

5 $$\begin{aligned} V\left( z \right) & > 0 \\ \dot{V}\left( z \right) & \le - 2^{{\frac{{1 + \mu }}{2}}} pV\left( z \right)^{{\frac{{1 + \mu }}{2}}} - 2qV\left( z \right),\forall z \in U\backslash \left\{ 0 \right\} \\ \end{aligned}$$

then, the origin of system (4) is finite-time stable. The settling time can be obtained from the initial state

6 $$T\left( {z\left( 0 \right)} \right) \le \frac{{\ln \left( {1 + 2^{{\frac{1 - \mu }{2}}} \frac{q}{p}V\left( {z\left( 0 \right)} \right)^{1 - \mu } } \right)}}{{q\left( {1 - \mu } \right)}}$$

and $T$ is continuous on $U$. Furthermore, if $D = {\varvec{R}}^{n}$, and $V > 0$, $\dot{V} < 0$ on ${\varvec{R}}^{n} \backslash \left\{ 0 \right\}$, then the origin of system (4) is globally finite-time stable.

## Main results

This section will propose two adaptive feedback hybrid control methods for finite-time synchronization of different dimensional chaotic systems with uncertain parameters and external disturbances.

Because the external disturbance functions $h_{i} \left( t \right)(i = 1,2, \ldots ,n)$ and $H_{j} \left( t \right)(j = 1,2, \ldots ,m)$ are bounded, there are constants $d_{i} (i = 1,2, \ldots ,n)$ and $D_{j} (i = 1,2, \ldots ,m)$ such that

7 $$\left| {h_{i} \left( t \right)} \right| \le d_{i} ,\;\;\left| {H_{j} \left( t \right)} \right| \le D_{j}$$

For

8 $$\begin{aligned} \left( {\Theta \user2{H}\left( t \right) - \Phi \user2{h}\left( t \right)} \right)\left( i \right) & = \left( \Theta \right)\left( i \right)\user2{H}\left( t \right) - \left( \Phi \right)\left( i \right)\user2{h}\left( t \right) \\ & = \sum\limits_{{j = 1}}^{m} {\left( \Theta \right)\left( {ij} \right)H_{j} \left( t \right)} - \sum\limits_{{j = 1}}^{n} {\left( \Phi \right)\left( {ij} \right)h_{j} \left( t \right)} \\ \end{aligned}$$

therefore

9 $$\begin{aligned} \left| {\left( {\Theta \user2{H}\left( t \right) - \Phi \user2{h}\left( t \right)} \right)\left( i \right)} \right| & = \left| {\sum\limits_{{j = 1}}^{m} {\left( \Theta \right)\left( {ij} \right)H_{j} \left( t \right)} - \sum\limits_{{j = 1}}^{n} {\left( \Phi \right)\left( {ij} \right)h_{j} \left( t \right)} } \right| \\ & \le \left| {\sum\limits_{{j = 1}}^{m} {\left( \Theta \right)\left( {ij} \right)} } \right|D_{j} + \left| {\sum\limits_{{j = 1}}^{n} {\left( \Phi \right)\left( {ij} \right)} } \right|d_{j} \\ \end{aligned}$$

Denote

10 $$d_{i}^{ * } = \left| {\sum\limits_{j = 1}^{m} {\left( \Theta \right)\left( {ij} \right)} } \right|D_{j} + \left| {\sum\limits_{j = 1}^{n} {\left( \Phi \right)\left( {ij} \right)} } \right|d_{j}$$

for $\Theta \left( {ij} \right),\Phi \left( {ij} \right)$ are constant, $h_{i} \left( t \right)$ and $H_{j} \left( t \right)$ are bounded, so $d_{i}^{ * }$ is also bounded.

To sum up, we can draw a conclusion

11 $$\left| {\left( {\Theta {\varvec{H}}\left( t \right) - \Phi {\varvec{h}}\left( t \right)} \right)\left( i \right)} \right| \le d_{i}^{ * }$$

where $d_{i} ,D_{j} \left( {i = 1,2, \ldots ,n,j = 1,2, \ldots ,m} \right)$ are constant values.

The error dynamic system is given as follows:

12 $$\begin{aligned} \user2{\dot{e}} & = \Theta \user2{\dot{y}} - \Phi \user2{\dot{x}} \\ & = \Theta g\left( \user2{y} \right)\user2{\beta - }\Phi f\left( \user2{x} \right)\user2{\alpha } + \Theta G\left( \user2{y} \right) - \Phi F\left( \user2{x} \right) + \Theta \user2{H}\left( t \right) - \Phi \user2{h}\left( t \right) + \Theta \user2{u} \\ \end{aligned}$$

Denote

13 $$R_{1} = \Theta G\left( {\varvec{y}} \right) - \Phi F\left( {\varvec{x}} \right) + \Theta g\left( {\varvec{y}} \right)\hat{\user2{\beta }} - \Phi f\left( {\varvec{x}} \right)\hat{\user2{\alpha }} + S\left( {\varvec{e}} \right)\hat{\user2{d}} + q_{1} \cdot {\varvec{e}} + p_{1} \cdot D\left( {\left| {\varvec{e}} \right|^{{\mu_{1} }} } \right)S\left( {\varvec{e}} \right)$$

where $\Theta \in {\varvec{R}}^{m \times m} ,\Phi \in {\varvec{R}}^{m \times n}$ are scaling constant matrices,$\hat{\user2{\alpha }},\hat{\user2{\beta }},\hat{\user2{d}}$ are the adaptive estimations of parameters ${\varvec{\alpha}},{\varvec{\beta}},{\varvec{d}}^{ * }$, respectively, ${\varvec{d}}^{ * } = \left( {d_{1}^{ * } ,d_{2}^{ * } , \ldots ,d_{m}^{ * } } \right)$ is a constants vector, and $S\left( {\varvec{e}} \right) = \left( {sign\left( {e_{1} } \right), \ldots ,sign\left( {e_{m} } \right)} \right)^{T}$, $D\left( {\varvec{e}} \right) = diag\left( {e_{1} , \ldots ,e_{m} } \right)$, $D\left( {\left| {\varvec{e}} \right|^{{\mu_{1} }} } \right) = diag\left( {\left| {e_{1} } \right|{}^{{\mu_{1} }}, \ldots ,\left| {e_{m} } \right|{}^{{\mu_{1} }}} \right)$, $D\left( {\left| {\varvec{e}} \right|^{{\mu_{2} }} } \right) = diag\left( {\left| {e_{1} } \right|{}^{{\mu_{2} }}, \ldots ,\left| {e_{m} } \right|{}^{{\mu_{2} }}} \right)$, $0 < \mu_{1},u_{2} < 1$, $p_{1}$ and $q_{1}$ are control gain constants, all positive real numbers.

Then, based on the adaptive control method^22^, the controller in Eq. (2) is designed as follows

14 $${\varvec{u}} = - \Theta^{ - 1} R_{1}$$

The following adaptive laws are proposed to estimate the unknown parameters

15 $$\begin{aligned} \Delta \user2{\dot{d}} & = - D\left( \user2{e} \right)S\left( \user2{e} \right) - p_{1} \cdot D\left( {\left| {\Delta \user2{d}} \right|^{{\mu _{1} }} } \right)S\left( {\Delta \user2{d}} \right) - q_{1} \left( {\Delta \user2{d}} \right) \\ \Delta \user2{\dot{\alpha }} & = \left( {\Phi f\left( \user2{x} \right)} \right)^{T} \user2{e} - p_{1} \cdot D\left( {\left| {\Delta \user2{\alpha }} \right|^{{\mu _{1} }} } \right)S\left( {\Delta \user2{\alpha }} \right) - q_{1} \left( {\Delta \user2{\alpha }} \right) \\ \Delta \user2{\dot{\beta }} & = - \left( {\Theta g\left( \user2{y} \right)} \right)^{T} \user2{e} - p_{1} \cdot D\left( {\left| {\Delta \user2{\beta }} \right|^{{\mu _{1} }} } \right)S\left( {\Delta \user2{\beta }} \right) - q_{1} \left( {\Delta \user2{\beta }} \right) \\ \end{aligned}$$

where $$\Delta {\varvec{\alpha}} = {\varvec{\alpha}} - \hat{\user2{\alpha }}$$, $$\Delta {\varvec{\beta}} = {\varvec{\beta}} - \hat{\user2{\beta }}$$, $$\Delta {\varvec{d}} = {\varvec{d}}^{ * } - \hat{\user2{d}}$$, and $$S\left( {\Delta {\varvec{d}}} \right) = \left( {sign\left( {\Delta d_{1} } \right), \ldots ,sign\left( {\Delta d_{m} } \right)} \right)^{T}$$,

$$S\left( {\Delta {\varvec{\alpha}}} \right) = \left( {sign\left( {\Delta \alpha_{1} } \right), \ldots ,sign\left( {\Delta \alpha_{{\tau_{1} }} } \right)} \right)^{T}$$, $$S\left( {\Delta {\varvec{\beta}}} \right) = \left( {sign\left( {\Delta \beta_{1} } \right), \ldots ,sign\left( {\Delta \beta_{{\tau_{2} }} } \right)} \right)^{T}$$,

$$D\left( {\left| {\Delta {\varvec{d}}} \right|^{{\mu_{1} }} } \right) = diag\left( {\left| {\Delta d_{1} } \right|{}^{{\mu_{1} }}, \ldots ,\left| {\Delta d_{m} } \right|{}^{{\mu_{1} }}} \right)$$, $$D\left( {\left| {\Delta {\varvec{\alpha}}} \right|^{{\mu_{1} }} } \right) = diag\left( {\left| {\Delta \alpha_{1} } \right|{}^{{\mu_{1} }}, \ldots ,\left| {\Delta \alpha_{{\tau_{1} }} } \right|{}^{{\mu_{1} }}} \right)$$ and

$$D\left( {\left| {\Delta {\varvec{\beta}}} \right|^{{\mu_{1} }} } \right) = diag\left( {\left| {\Delta \beta_{1} } \right|{}^{{\mu_{1} }}, \ldots ,\left| {\Delta \beta_{{\tau_{2} }} } \right|{}^{{\mu_{1} }}} \right)$$.

### Theorem 1.

For chaotic or hyperchaotic systems (1) and (2), if they satisfy the assumptions (1) to (3), for any initial values ${\varvec{x}}\left( 0 \right),{\varvec{y}}\left( 0 \right)$ and given scaling matrices $\Theta$ and $\Phi$, the systems can achieve finite-time synchronization by the controller (14) and the parameter adaptive laws (15) with the settling time, given by $T_{1} = \frac{{\ln \left( {1 + 2^{{\frac{{1 - \mu_{1} }}{2}}} \frac{{q_{1} }}{{p_{1} }}V\left( 0 \right)^{{\frac{{1 - \mu_{1} }}{2}}} } \right)}}{{q_{1} \left( {1 - \mu_{1} } \right)}}$.

### Proof

Substituting (14) into (12), we have

16 $$\dot{\user2{e}} = \Theta g\left( {\varvec{y}} \right)\Delta {\varvec{\beta}} - \Phi f\left( {\varvec{x}} \right)\Delta {\varvec{\alpha}} + \Theta {\varvec{H}}\left( t \right) - \Phi {\varvec{h}}\left( t \right) - S\left( {\varvec{e}} \right)\hat{\user2{d}} - p_{1} \cdot D\left( {\left| {\varvec{e}} \right|^{{\mu_{1} }} } \right)S\left( {\varvec{e}} \right) - q_{1} \cdot {\varvec{e}}$$

We introduce the following Lyapunov function

17 $$V = \frac{1}{2}\left( {{\varvec{e}}^{T} {\varvec{e}} + \left( {\Delta {\varvec{\beta}}} \right)^{T} \Delta {\varvec{\beta}} + \left( {\Delta {\varvec{\alpha}}} \right)^{T} \Delta {\varvec{\alpha}} + \left( {\Delta {\varvec{d}}} \right)^{T} \left( {\Delta {\varvec{d}}} \right)} \right)$$

Take the derivative of $V\left( t \right)$ along Eqs. (15) and (16)

18 $$\begin{aligned} \dot{V} & = \user2{e}^{T} \user2{\dot{e}} + \left( {\Delta \user2{\beta }} \right)^{T} \Delta \user2{\dot{\beta }} + \left( {\Delta \user2{\alpha }} \right)^{T} \Delta \user2{\dot{\alpha }} + \left( {\Delta \user2{d}} \right)^{T} \Delta \user2{\dot{d}} \\ & = \user2{e}^{T} \left( {\Theta \user2{H}\left( t \right) - \Phi \user2{h}\left( t \right)} \right) - \user2{e}^{T} S\left( \user2{e} \right)\user2{\hat{d}} - p_{1} \cdot \user2{e}^{T} D\left( {\left| \user2{e} \right|^{{\mu _{1} }} } \right)S\left( \user2{e} \right) - q_{1} \cdot \user2{e}^{T} \user2{e} \\ & \;\; - p_{1} \cdot \left( {\Delta \user2{\beta }} \right)^{T} D\left( {\left| {\Delta \user2{\beta }} \right|^{{\mu _{1} }} } \right)S\left( {\Delta \user2{\beta }} \right) - q_{1} \left( {\Delta \user2{\beta }} \right)^{T} \left( {\Delta \user2{\beta }} \right) - p_{1} \cdot \left( {\Delta \user2{\alpha }} \right)^{T} D\left( {\left| {\Delta \user2{\alpha }} \right|^{{\mu _{1} }} } \right)S\left( {\Delta \user2{\alpha }} \right) - q_{1} \left( {\Delta \user2{\alpha }} \right)^{T} \left( {\Delta \user2{\alpha }} \right) \\ & \;\;{\text{ + }}\left( {\user2{d}^{ * } - \user2{\hat{d}}} \right)^{T} \left( { - D\left( \user2{e} \right)S\left( \user2{e} \right) - p_{1} \cdot D\left( {\left| {\Delta \user2{d}} \right|^{{\mu _{1} }} } \right)S\left( {\Delta \user2{d}} \right) - q_{1} \left( {\Delta \user2{d}} \right)} \right) \\ & \le \user2{e}^{T} \user2{d}^{ * } - \user2{e}^{T} S\left( \user2{e} \right)\user2{\hat{d}} - p_{1} \cdot \user2{e}^{T} D\left( {\left| \user2{e} \right|^{{\mu _{1} }} } \right)S\left( \user2{e} \right) - q_{1} \cdot \user2{e}^{T} \user2{e} - p_{1} \cdot \left( {\Delta \user2{\beta }} \right)^{T} D\left( {\left| {\Delta \user2{\beta }} \right|^{{\mu _{1} }} } \right)S\left( {\Delta \user2{\beta }} \right) - q_{1} \left( {\Delta \user2{\beta }} \right)^{T} \left( {\Delta \user2{\beta }} \right) \\ & \;\; - p_{1} \cdot \left( {\Delta \user2{\alpha }} \right)^{T} D\left( {\left| {\Delta \user2{\alpha }} \right|^{{\mu _{1} }} } \right)S\left( {\Delta \user2{\alpha }} \right) - q_{1} \left( {\Delta \user2{\alpha }} \right)^{T} \left( {\Delta \user2{\alpha }} \right) \\ & \;\;{\text{ + }}\left( {\user2{d}^{ * } - \user2{\hat{d}}} \right)^{T} \left( { - D\left( \user2{e} \right)S\left( \user2{e} \right) - p_{1} \cdot D\left( {\left| {\Delta \user2{d}} \right|^{{\mu _{1} }} } \right)S\left( {\Delta \user2{d}} \right) - q_{1} \left( {\Delta \user2{d}} \right)} \right) \\ & \le - q_{1} \cdot \user2{e}^{T} \user2{e} - q_{1} \left( {\Delta \user2{\beta }} \right)^{T} \left( {\Delta \user2{\beta }} \right) - q_{1} \left( {\Delta \user2{\alpha }} \right)^{T} \left( {\Delta \user2{\alpha }} \right) - q_{1} \left( {\Delta \user2{d}} \right)^{T} \left( {\Delta \user2{d}} \right) - p_{1} \cdot \user2{e}^{T} D\left( {\left| \user2{e} \right|^{{\mu _{1} }} } \right)S\left( \user2{e} \right) \\ & \; - p_{1} \cdot \left( {\Delta \user2{\beta }} \right)^{T} D\left( {\left| {\Delta \user2{\beta }} \right|^{{\mu _{1} }} } \right)S\left( {\Delta \user2{\beta }} \right) - p_{1} \cdot \left( {\Delta \user2{\alpha }} \right)^{T} D\left( {\left| {\Delta \user2{\alpha }} \right|^{{\mu _{1} }} } \right)S\left( {\Delta \user2{\alpha }} \right) - p_{1} \cdot \left( {\Delta \user2{d}} \right)^{T} D\left( {\left| {\Delta \user2{d}} \right|^{{\mu _{1} }} } \right)S\left( {\Delta \user2{d}} \right) \\ & \; < 0 \\ \end{aligned}$$

Because $0 < \mu_{1} < 1$, then $\frac{1}{2} < \frac{{1 + \mu_{1} }}{2} < 1$, there are

19 $$\begin{aligned} \dot{V} & = \user2{e}^{T} \user2{\dot{e}} + \left( {\Delta \user2{\beta }} \right)^{T} \Delta \user2{\dot{\beta }} + \left( {\Delta \user2{\alpha }} \right)^{T} \Delta \user2{\dot{\alpha }} + \left( {\Delta \user2{d}} \right)^{T} \Delta \user2{\dot{d}} \\ & \le - q_{1} \cdot \user2{e}^{T} \user2{e} - q_{1} \left( {\Delta \user2{\beta }} \right)^{T} \left( {\Delta \user2{\beta }} \right) - q_{1} \left( {\Delta \user2{\alpha }} \right)^{T} \left( {\Delta \user2{\alpha }} \right) - q_{1} \left( {\Delta \user2{d}} \right)^{T} \left( {\Delta \user2{d}} \right) - p_{1} \cdot \user2{e}^{T} D\left( {\left| \user2{e} \right|^{{\mu _{1} }} } \right)S\left( \user2{e} \right) \\ & \;\; - p_{1} \cdot \left( {\Delta \user2{\beta }} \right)^{T} D\left( {\left| {\Delta \user2{\beta }} \right|^{{\mu _{1} }} } \right)S\left( {\Delta \user2{\beta }} \right) - p_{1} \cdot \left( {\Delta \user2{\alpha }} \right)^{T} D\left( {\left| {\Delta \user2{\alpha }} \right|^{{\mu _{1} }} } \right)S\left( {\Delta \user2{\alpha }} \right) - p_{1} \cdot \left( {\Delta \user2{d}} \right)^{T} D\left( {\left| {\Delta \user2{d}} \right|^{{\mu _{1} }} } \right)S\left( {\Delta \user2{d}} \right) \\ & = - p_{1} \cdot \left( {\user2{e}^{T} D\left( {\left| \user2{e} \right|^{{\mu _{1} }} } \right)S\left( \user2{e} \right) + \left( {\Delta \user2{\beta }} \right)^{T} D\left( {\left| {\Delta \user2{\beta }} \right|^{{\mu _{1} }} } \right)S\left( {\Delta \user2{\beta }} \right) + \left( {\Delta \user2{\alpha }} \right)^{T} D\left( {\left| {\Delta \user2{\alpha }} \right|^{{\mu _{1} }} } \right)S\left( {\Delta \user2{\alpha }} \right) + \left( {\Delta \user2{d}} \right)^{T} D\left( {\left| {\Delta \user2{d}} \right|^{{\mu _{1} }} } \right)S\left( {\Delta \user2{d}} \right)} \right) \\ & \;\; - q_{1} \left( {\user2{e}^{T} \user2{e} + \left( {\Delta \user2{\beta }} \right)^{T} \left( {\Delta \user2{\beta }} \right) + \left( {\Delta \user2{\alpha }} \right)^{T} \left( {\Delta \user2{\alpha }} \right) + \left( {\Delta \user2{d}} \right)^{T} \left( {\Delta \user2{d}} \right)} \right) \\ & \le - p_{1} \cdot \left( {\left( {\user2{e}^{T} \user2{e}} \right)^{{\frac{{1 + \mu _{1} }}{2}}} + \left( {\left( {\Delta \user2{\beta }} \right)^{T} \left( {\Delta \user2{\beta }} \right)} \right)^{{\frac{{1 + \mu _{1} }}{2}}} + \left( {\left( {\Delta \user2{\alpha }} \right)^{T} \left( {\Delta \user2{\alpha }} \right)} \right)^{{\frac{{1 + \mu _{1} }}{2}}} + \left( {\left( {\Delta \user2{d}} \right)^{T} \left( {\Delta \user2{d}} \right)} \right)^{{\frac{{1 + \mu _{1} }}{2}}} } \right) \\ & \;\; - q_{1} \cdot \left( {\user2{e}^{T} \user2{e} + \left( {\Delta \user2{\beta }} \right)^{T} \left( {\Delta \user2{\beta }} \right) + \left( {\Delta \user2{\alpha }} \right)^{T} \left( {\Delta \user2{\alpha }} \right) + \left( {\Delta \user2{d}} \right)^{T} \left( {\Delta \user2{d}} \right)} \right) \\ & \le - p_{1} \cdot \left( {\user2{e}^{T} \user2{e} + \left( {\Delta \user2{\beta }} \right)^{T} \left( {\Delta \user2{\beta }} \right) + \left( {\Delta \user2{\alpha }} \right)^{T} \left( {\Delta \user2{\alpha }} \right) + \left( {\Delta \user2{d}} \right)^{T} \left( {\Delta \user2{d}} \right)} \right)^{{\frac{{1 + \mu _{1} }}{2}}} \\ & \;\; - q_{1} \cdot \left( {\user2{e}^{T} \user2{e} + \left( {\Delta \user2{\beta }} \right)^{T} \left( {\Delta \user2{\beta }} \right) + \left( {\Delta \user2{\alpha }} \right)^{T} \left( {\Delta \user2{\alpha }} \right) + \left( {\Delta \user2{d}} \right)^{T} \left( {\Delta \user2{d}} \right)} \right) \\ & = - 2^{{\frac{{1 + \mu _{1} }}{2}}} p_{1} \cdot V^{{\frac{{1 + \mu _{1} }}{2}}} - 2q_{1} \cdot V \\ \end{aligned}$$

From Eqs. (17) and (18), it can be seen that $V > 0$ and $\dot{V} < 0$ near the origin, according to Lyapunov stability theorem^23^ and Definition 1, the synchronization error system is asymptotically stable at the origin. Furthermore, from Eq. (19) and Proposition 1, the error system (16) is stable for a finite time at the origin, and the stability time $T_{1} = \frac{{\ln \left( {1 + 2^{{\frac{{1 - \mu_{1} }}{2}}} \frac{{q_{1} }}{{p_{1} }}V\left( 0 \right)^{{\frac{{1 - \mu_{1} }}{2}}} } \right)}}{{q_{1} \left( {1 - \mu_{1} } \right)}}$, that is, the driving system (1) and the response system (2) can achieve finite-time synchronization.

The proof is completed.

### Remark 2.

Let denote $\alpha_{i}$ or $\beta_{i}$ be $i$-th unknown parameter of $i$-th equation in the system.

### Assumption 4.

Systems (1) and (2) are linear at an unknown parameter $\alpha_{i}$ or $\beta_{i}$.

When systems (1) and (2) satisfy Assumption 4, then they can be written as

20 $$\begin{aligned} \user2{\dot{x}} & = A_{\user2{\alpha }} \user2{x} + F\left( \user2{x} \right) \\ \user2{\dot{y} } & {\text{ = }}B_{\user2{\beta }} \user2{y} + G\left( \user2{y} \right) + \user2{u} \\ \end{aligned}$$

where $$A_{{\varvec{\alpha}}} { = }diag\left( {\alpha_{1} , \ldots ,\alpha_{n} } \right)$$, $$B_{{\varvec{\beta}}} { = }diag\left( {\beta_{1} , \ldots ,\beta_{m} } \right)$$.

Denote

21 $$\tilde{\user2{e}} = \Omega {\varvec{e}}$$

where $\Omega = \left( {\Omega_{ij} } \right) \in {\mathbf{R}}^{n \times m}$ is the transformation matrix,$\tilde{\user2{e}} = \left( {e_{1} , \ldots ,e_{n} } \right)^{T} ,{\varvec{e}} = \left( {e_{1} , \ldots ,e_{m} } \right)^{T}$. If $n \le m$, then $$\Omega_{ij} = \left\{ \begin{gathered} \begin{array}{*{20}l} 1 & {i = j} \\ \end{array} \hfill \\ \begin{array}{*{20}l} 0 & {others} \\ \end{array} \hfill \\ \end{gathered} \right.$$; if $n > m$, then $$\Omega_{ij} = \left\{ \begin{gathered} \begin{array}{*{20}l} 1 & {i = j,i = 1, \ldots ,m} \\ \end{array} \hfill \\ \begin{array}{*{20}l} 1 & {j = m,i = m + 1, \ldots ,n} \\ \end{array} \hfill \\ \begin{array}{*{20}l} 0 & {others} \\ \end{array} \hfill \\ \end{gathered} \right.$$.

The error dynamic system is obtained as

22 $$\begin{aligned} \user2{\dot{e}} & = \Theta \user2{\dot{y}} - \Phi \user2{\dot{x}} \\ & = \Theta B_{\user2{\beta }} \user2{y} - \Phi A_{\user2{\alpha }} \user2{x} + \Theta G\left( \user2{y} \right) - \Phi F\left( \user2{x} \right) + \Theta \user2{H}\left( t \right) - \Phi \user2{h}\left( t \right) + \Theta \user2{u} \\ \end{aligned}$$

Denote

23 $$R_{2} = \Theta \left( {B_{{\varvec{\beta}}} {\varvec{y}} + G\left( {\varvec{y}} \right) - B_{{\Delta {\varvec{\beta}}}} {\varvec{e}}} \right) + \Phi A_{{\Delta {\varvec{\alpha}}}} \tilde{\user2{e}} - \Phi A_{{\varvec{\alpha}}} {\varvec{x}} - \Phi F\left( {\varvec{x}} \right) + S\left( {\varvec{e}} \right)\hat{\user2{d}} + q_{2} {\varvec{e}} + p_{2} \cdot D\left( {\left| {\varvec{e}} \right|^{{\mu_{2} }} } \right)S\left( {\varvec{e}} \right)$$

where $$A_{{\Delta {\varvec{\alpha}}}} = A_{{\varvec{\alpha}}} - A_{{\hat{\user2{\alpha }}}} = diag\left( {\Delta \alpha_{1} , \ldots ,\Delta \alpha_{n} } \right)$$, $$B_{{\Delta {\varvec{\beta}}}} = B_{{\varvec{\beta}}} - B_{{\hat{\user2{\beta }}}} = diag\left( {\Delta \beta_{1} , \ldots ,\Delta \beta_{m} } \right)$$.

Then, to achieve systems synchronization, based on the adaptive control principle, the controller in Eq. (20) is designed as follows

24 $${\varvec{u}} = - \Theta^{ - 1} R_{2}$$

The following adaptive laws are proposed to estimate the unknown parameters

25 $$\begin{aligned} \Delta \user2{\dot{d}} & = - D\left( \user2{e} \right)S\left( \user2{e} \right) - p_{2} \cdot D\left( {\left| {\Delta \user2{d}} \right|^{{\mu _{2} }} } \right)S\left( {\Delta \user2{d}} \right) - q_{2} \left( {\Delta \user2{d}} \right) \\ \Delta \user2{\dot{\alpha }} & = D\left( {\user2{\tilde{e}}} \right)\Phi ^{T} \user2{\tilde{e}} - p_{2} \cdot D\left( {\left| {\Delta \user2{\alpha }} \right|^{{\mu _{2} }} } \right)S\left( {\Delta \user2{\alpha }} \right) - q_{2} \left( {\Delta \user2{\alpha }} \right) \\ \Delta \user2{\dot{\beta }} & = - D\left( \user2{e} \right)\Theta ^{T} \user2{e} - p_{2} \cdot D\left( {\left| {\Delta \user2{\beta }} \right|^{{\mu _{2} }} } \right)S\left( {\Delta \user2{\beta }} \right) - q_{2} \left( {\Delta \user2{\beta }} \right) \\ \end{aligned}$$

### Theorem 2.

For chaotic or hyperchaotic systems, if their expressions can be expressed as (23) and satisfy the assumptions (1) to (4), for any initial value ${\varvec{x}}\left( 0 \right),{\varvec{y}}\left( 0 \right)$ and given scaling matrices $\Theta$ and $\Phi$, the systems can achieve finite-time synchronization by the controller (24) and the parameter adaptive laws (25) with the settling time, given by $T_{2} = \frac{{\ln \left( {1 + 2^{{\frac{{1 - \mu_{2} }}{2}}} \frac{{q_{2} }}{{p_{2} }}V\left( 0 \right)^{{\frac{{1 - \mu_{2} }}{2}}} } \right)}}{{q_{2} \left( {1 - \mu_{2} } \right)}}$.

**Proof**. Substituting (24) into (22), we have

26 $$\dot{\user2{e}} = \Theta B_{{\Delta {\varvec{\beta}}}} {\varvec{e}} - \Phi A_{{\Delta {\varvec{\alpha}}}} \tilde{\user2{e}} + \Theta {\varvec{H}}\left( t \right) - \Phi {\varvec{h}}\left( t \right) - S\left( {\varvec{e}} \right)\hat{\user2{d}} - p_{2} \cdot D\left( {\left| {\varvec{e}} \right|^{{\mu_{2} }} } \right)S\left( {\varvec{e}} \right) - q_{2} {\varvec{e}}$$

We introduce the following Lyapunov function

27 $$V = \frac{1}{2}\left( {{\varvec{e}}^{T} {\varvec{e}} + \left( {\Delta {\varvec{\beta}}} \right)^{T} \Delta {\varvec{\beta}} + \left( {\Delta {\varvec{\alpha}}} \right)^{T} \Delta {\varvec{\alpha}} + \left( {\Delta {\varvec{d}}} \right)^{T} \left( {\Delta {\varvec{d}}} \right)} \right)$$

Take the derivative of $V\left( t \right)$ along Eqs. (25) and (26)

28 $$\begin{aligned} \dot{V} & = \user2{e}^{T} \user2{\dot{e}} + \left( {\Delta \user2{\beta }} \right)^{T} \Delta \user2{\dot{\beta }} + \left( {\Delta \user2{\alpha }} \right)^{T} \Delta \user2{\dot{\alpha }} + \left( {\Delta \user2{d}} \right)^{T} \Delta \user2{\dot{d}} \\ & = \user2{e}^{T} \left( {\user2{H}\left( t \right) - \user2{h}\left( t \right)} \right) - S\left( \user2{e} \right)\user2{\hat{d}} - p_{2} \cdot \user2{e}^{T} D\left( {\left| \user2{e} \right|^{{\mu _{2} }} } \right)S\left( \user2{e} \right) - q_{2} \cdot \user2{e}^{T} \user2{e} \\ & \;\; - p_{2} \cdot \left( {\Delta \user2{\beta }} \right)^{T} D\left( {\left| {\Delta \user2{\beta }} \right|^{{\mu _{2} }} } \right)S\left( {\Delta \user2{\beta }} \right) - q_{2} \left( {\Delta \user2{\beta }} \right)^{T} \left( {\Delta \user2{\beta }} \right) \\ & \;\; - p_{2} \cdot \left( {\Delta \user2{\alpha }} \right)^{T} D\left( {\left| {\Delta \user2{\alpha }} \right|^{{\mu _{2} }} } \right)S\left( {\Delta \user2{\alpha }} \right) - q_{2} \left( {\Delta \user2{\alpha }} \right)^{T} \left( {\Delta \user2{\alpha }} \right) \\ & \;\;{\text{ + }}\left( {\user2{d}^{ * } - \user2{\hat{d}}} \right)^{T} \left( { - D\left( \user2{e} \right)S\left( \user2{e} \right) - p_{2} \cdot D\left( {\left| {\Delta \user2{d}} \right|^{{\mu _{2} }} } \right)S\left( {\Delta \user2{d}} \right) - q_{2} \left( {\Delta \user2{d}} \right)} \right) \\ & \le \user2{e}^{T} \user2{d}^{ * } - \user2{e}^{T} S\left( \user2{e} \right)\user2{\hat{d}} - p_{2} \cdot \user2{e}^{T} D\left( {\left| \user2{e} \right|^{{\mu _{2} }} } \right)S\left( \user2{e} \right) - q_{2} \cdot \user2{e}^{T} \user2{e} \\ & \;\; - p_{2} \cdot \left( {\Delta \user2{\beta }} \right)^{T} D\left( {\left| {\Delta \user2{\beta }} \right|^{{\mu _{2} }} } \right)S\left( {\Delta \user2{\beta }} \right) - q_{2} \left( {\Delta \user2{\beta }} \right)^{T} \left( {\Delta \user2{\beta }} \right) \\ & \;\; - p_{2} \cdot \left( {\Delta \user2{\alpha }} \right)^{T} D\left( {\left| {\Delta \user2{\alpha }} \right|^{{\mu _{2} }} } \right)S\left( {\Delta \user2{\alpha }} \right) - q_{2} \left( {\Delta \user2{\alpha }} \right)^{T} \left( {\Delta \user2{\alpha }} \right) \\ & \;\;{\text{ + }}\left( {\user2{d}^{ * } - \user2{\hat{d}}} \right)^{T} \left( { - D\left( \user2{e} \right)S\left( \user2{e} \right) - p_{2} \cdot D\left( {\left| {\Delta \user2{d}} \right|^{{\mu _{2} }} } \right)S\left( {\Delta \user2{d}} \right) - q_{2} \left( {\Delta \user2{d}} \right)} \right) \\ & \le - q_{2} \cdot \user2{e}^{T} \user2{e} - q_{2} \left( {\Delta \user2{\beta }} \right)^{T} \left( {\Delta \user2{\beta }} \right) - q_{2} \left( {\Delta \user2{\alpha }} \right)^{T} \left( {\Delta \user2{\alpha }} \right) - q_{2} \left( {\Delta \user2{d}} \right)^{T} \left( {\Delta \user2{d}} \right) \\ & \;\; - p_{2} \cdot \user2{e}^{T} D\left( {\left| \user2{e} \right|^{{\mu _{2} }} } \right)S\left( \user2{e} \right) - p_{2} \cdot \left( {\Delta \user2{\beta }} \right)^{T} D\left( {\left| {\Delta \user2{\beta }} \right|^{{\mu _{2} }} } \right)S\left( {\Delta \user2{\beta }} \right) \\ & \;\; - p_{2} \cdot \left( {\Delta \user2{\alpha }} \right)^{T} D\left( {\left| {\Delta \user2{\alpha }} \right|^{{\mu _{2} }} } \right)S\left( {\Delta \user2{\alpha }} \right) - p_{2} \cdot \left( {\Delta \user2{d}} \right)^{T} D\left( {\left| {\Delta \user2{d}} \right|^{{\mu _{2} }} } \right)S\left( {\Delta \user2{d}} \right) \\ & \; < 0 \\ \end{aligned}$$

Because $0 < \mu_{2} < 1$, then $\frac{1}{2} < \frac{{1 + \mu_{2} }}{2} < 1$, there are

29 $$\begin{aligned} \dot{V} & = \user2{e}^{T} \user2{\dot{e}} + \left( {\Delta \user2{\beta }} \right)^{T} \Delta \user2{\dot{\beta }} + \left( {\Delta \user2{\alpha }} \right)^{T} \Delta \user2{\dot{\alpha }} + \left( {\Delta \user2{d}} \right)^{T} \Delta \user2{\dot{d}} \\ & \le - q_{2} \cdot \user2{e}^{T} \user2{e} - q_{2} \left( {\Delta \user2{\beta }} \right)^{T} \left( {\Delta \user2{\beta }} \right) - q_{2} \left( {\Delta \user2{\alpha }} \right)^{T} \left( {\Delta \user2{\alpha }} \right) - q_{2} \left( {\Delta \user2{d}} \right)^{T} \left( {\Delta \user2{d}} \right) - p_{2} \cdot \user2{e}^{T} D\left( {\left| \user2{e} \right|^{{\mu _{2} }} } \right)S\left( \user2{e} \right) \\ & \;\; - p_{2} \cdot \left( {\Delta \user2{\beta }} \right)^{T} D\left( {\left| {\Delta \user2{\beta }} \right|^{{\mu _{2} }} } \right)S\left( {\Delta \user2{\beta }} \right) - p_{2} \cdot \left( {\Delta \user2{\alpha }} \right)^{T} D\left( {\left| {\Delta \user2{\alpha }} \right|^{{\mu _{2} }} } \right)S\left( {\Delta \user2{\alpha }} \right) \\ & \;\; - p_{2} \cdot \left( {\Delta \user2{d}} \right)^{T} D\left( {\left| {\Delta \user2{d}} \right|^{{\mu _{2} }} } \right)S\left( {\Delta \user2{d}} \right) \\ & = - p_{2} \cdot \left( {\user2{e}^{T} D\left( {\left| \user2{e} \right|^{{\mu _{2} }} } \right)S\left( \user2{e} \right) + \left( {\Delta \user2{\beta }} \right)^{T} D\left( {\left| {\Delta \user2{\beta }} \right|^{{\mu _{2} }} } \right)S\left( {\Delta \user2{\beta }} \right) + \left( {\Delta \user2{\alpha }} \right)^{T} D\left( {\left| {\Delta \user2{\alpha }} \right|^{{\mu _{2} }} } \right)S\left( {\Delta \user2{\alpha }} \right) + \left( {\Delta \user2{d}} \right)^{T} D\left( {\left| {\Delta \user2{d}} \right|^{{\mu _{2} }} } \right)S\left( {\Delta \user2{d}} \right)} \right) \\ & \;\; - q_{2} \cdot \left( {\user2{e}^{T} \user2{e} + \left( {\Delta \user2{\beta }} \right)^{T} \left( {\Delta \user2{\beta }} \right) + \left( {\Delta \user2{\alpha }} \right)^{T} \left( {\Delta \user2{\alpha }} \right) + \left( {\Delta \user2{d}} \right)^{T} \left( {\Delta \user2{d}} \right)} \right) \\ & \le - p_{2} \cdot \left( {\left( {\user2{e}^{T} \user2{e}} \right)^{{\frac{{1 + \mu _{2} }}{2}}} + \left( {\left( {\Delta \user2{\beta }} \right)^{T} \left( {\Delta \user2{\beta }} \right)} \right)^{{\frac{{1 + \mu _{2} }}{2}}} + \left( {\left( {\Delta \user2{\alpha }} \right)^{T} \left( {\Delta \user2{\alpha }} \right)} \right)^{{\frac{{1 + \mu _{2} }}{2}}} + \left( {\left( {\Delta \user2{d}} \right)^{T} \left( {\Delta \user2{d}} \right)} \right)^{{\frac{{1 + \mu _{2} }}{2}}} } \right) \\ & \;\; - q_{2} \cdot \left( {\user2{e}^{T} \user2{e} + \left( {\Delta \user2{\beta }} \right)^{T} \left( {\Delta \user2{\beta }} \right) + \left( {\Delta \user2{\alpha }} \right)^{T} \left( {\Delta \user2{\alpha }} \right) + \left( {\Delta \user2{d}} \right)^{T} \left( {\Delta \user2{d}} \right)} \right) \\ & \le - p_{2} \cdot \left( {\user2{e}^{T} \user2{e} + \left( {\Delta \user2{\beta }} \right)^{T} \left( {\Delta \user2{\beta }} \right) + \left( {\Delta \user2{\alpha }} \right)^{T} \left( {\Delta \user2{\alpha }} \right) + \left( {\Delta \user2{d}} \right)^{T} \left( {\Delta \user2{d}} \right)} \right)^{{\frac{{1 + \mu _{2} }}{2}}} \\ & \;\; - q_{2} \cdot \left( {\user2{e}^{T} \user2{e} + \left( {\Delta \user2{\beta }} \right)^{T} \left( {\Delta \user2{\beta }} \right) + \left( {\Delta \user2{\alpha }} \right)^{T} \left( {\Delta \user2{\alpha }} \right) + \left( {\Delta \user2{d}} \right)^{T} \left( {\Delta \user2{d}} \right)} \right) \\ & = - 2^{{\frac{{1 + \mu _{2} }}{2}}} p_{2} \cdot V^{{\frac{{1 + \mu _{2} }}{2}}} - 2q_{2} \cdot V \\ \end{aligned}$$

From Eqs. (27) and (28), it can be seen that $V > 0$ and $\dot{V} < 0$ near the origin, according to Definition 1, the synchronization error system is asymptotically stable at the origin. Furthermore, from Eq. (29) and Proposition 1, the error system (26) is stable for a finite time at the origin, and the stability time $T_{2} = \frac{{\ln \left( {1 + 2^{{\frac{{1 - \mu_{2} }}{2}}} \frac{{q_{2} }}{{p_{2} }}V\left( 0 \right)^{{\frac{{1 - \mu_{2} }}{2}}} } \right)}}{{q_{2} \left( {1 - \mu_{2} } \right)}}$, that is, the driving system and the response system (20) can achieve finite-time synchronization.

The proof is completed.

## Numerical simulations

### Example 1.

The Cai system is described as follows:

30 $$\left\{ \begin{gathered} \dot{x}_{1} = a_{1} \left( {x_{2} - x_{1} } \right) \hfill \\ \dot{x}_{2} = b_{1} x_{1} + c_{1} x_{2} - x_{1} x_{3} \hfill \\ \dot{x}_{3} = x_{1}^{2} - l_{1} x_{3} \hfill \\ \end{gathered} \right.$$

and the Rossler system is described as follows:

31 $$\left\{ \begin{gathered} \dot{y}_{1} = - y_{2} - y_{3} + u_{1} \hfill \\ \dot{y}_{2} = y_{1} + a_{2} y_{2} + y_{4} + u_{2} \hfill \\ \dot{y}_{3} = b_{2} + y{}_{1}y_{3} + u_{3} \hfill \\ \dot{y}_{4} = - c_{2} y_{3} + l_{2} y_{4} + u_{4} \hfill \\ \end{gathered} \right.$$

where the parameters are selected as $a_{1} = 20,b_{1} = 14,c_{1} = 10.6,l_{1} = 2.8$ and $a_{2} = 0.25,b_{2} = 3,c_{2} = 0.5,l_{2} = 0.05$, the system (30) and (31) are chaotic and hyperchaotic, respectively^24,25^. Rewrite the system Eqs. (30) and (31) with external disturbances and uncertain parameters into the following forms to synchronize them in four-dimensions.

32 $$\dot{\user2{x}} = f\left( {\varvec{x}} \right){\varvec{\alpha}} + F\left( {\varvec{x}} \right){ + }{\varvec{h}}\left( t \right)$$

where $$f\left( {\varvec{x}} \right){ = }\left[ {\begin{array}{*{20}l} {x_{2} - x_{1} } & 0 & 0 & 0 \\ 0 & {x_{1} } & {x_{2} } & 0 \\ 0 & 0 & 0 & { - x_{3} } \\ \end{array} } \right]$$, $${\varvec{\alpha}} = \left[ {\begin{array}{*{20}l} {a_{1} } \\ {b_{1} } \\ {c_{1} } \\ {l_{1} } \\ \end{array} } \right]$$, $$F\left( {\varvec{x}} \right) = \left[ {\begin{array}{*{20}l} 0 \\ { - x_{1} x_{3} } \\ {x_{1}^{2} } \\ \end{array} } \right]$$.

33 $$\dot{\user2{y}} = g\left( {\varvec{y}} \right){\varvec{\beta}} + G\left( {\varvec{y}} \right) + {\varvec{H}}\left( t \right) + {\varvec{u}}$$

where $$g\left( {\varvec{y}} \right) = \left[ {\begin{array}{*{20}l} 0 & 0 & 0 & 0 \\ {y_{2} } & 0 & 0 & 0 \\ 0 & 1 & 0 & 0 \\ 0 & 0 & { - y_{3} } & {y_{4} } \\ \end{array} } \right]$$, $${\varvec{\beta}} = \left[ {\begin{array}{*{20}l} {a_{2} } \\ {b_{2} } \\ {c_{2} } \\ {l_{2} } \\ \end{array} } \right]$$, $$G\left( {\varvec{x}} \right) = \left[ {\begin{array}{*{20}l} { - y_{2} - y_{3} } \\ {y_{1} + y_{4} } \\ {y_{1} y_{3} } \\ 0 \\ \end{array} } \right]$$, $${\varvec{u}} = \left[ \begin{gathered} u_{1} \hfill \\ u_{2} \hfill \\ u_{3} \hfill \\ u_{4} \hfill \\ \end{gathered} \right]$$.

We choose $$\Theta { = }\left[ {\begin{array}{*{20}l} 1 & 1 & 0 & 0 \\ 0 & 1 & 0 & 0 \\ 0 & 0 & 1 & 0 \\ 0 & 0 & 0 & 1 \\ \end{array} } \right],\Phi { = }\left[ {\begin{array}{*{20}l} 1 & 1 & 0 \\ 0 & 1 & 0 \\ 0 & 1 & 1 \\ 1 & 0 & 1 \\ \end{array} } \right]$$. From Eq. (15), the adaptive laws of parameters can be designed as follows

34 $$\begin{gathered} \left\{ \begin{gathered} \Delta \dot{a}_{1} = \left( {x_{2} - x_{1} } \right)\left( {e_{1} + e_{4} } \right) - p_{1} \cdot sign\left( {\Delta a_{1} } \right)\left| {\Delta a_{1} } \right|^{{\mu_{1} }} - q_{1} \cdot \left( {\Delta a_{1} } \right) \hfill \\ \Delta \dot{b}_{1} = x_{1} \left( {e_{1} + e_{2} + e_{3} } \right) - p_{1} \cdot sign\left( {\Delta b_{1} } \right)\left| {\Delta b_{1} } \right|^{{\mu_{1} }} - q_{1} \cdot \left( {\Delta b_{1} } \right) \hfill \\ \Delta \dot{c}_{1} = x_{2} \left( {e_{1} + e_{2} + e_{3} } \right) - p_{1} \cdot sign\left( {\Delta c_{1} } \right)\left| {\Delta c_{1} } \right|^{{\mu_{1} }} - q_{1} \cdot \left( {\Delta c_{1} } \right) \hfill \\ \Delta \dot{l}_{1} = - x_{3} \left( {e_{3} + e_{4} } \right) - p_{1} \cdot sign\left( {\Delta l_{1} } \right)\left| {\Delta l_{1} } \right|^{{\mu_{1} }} - q_{1} \cdot \left( {\Delta l_{1} } \right) \hfill \\ \end{gathered} \right. \hfill \\ \left\{ \begin{gathered} \Delta \dot{a}_{2} = - y_{2} \left( {e_{1} + e_{2} } \right) - p_{1} \cdot sign\left( {\Delta a_{2} } \right)\left| {\Delta a_{2} } \right|^{{\mu_{1} }} - q_{1} \cdot \left( {\Delta a_{2} } \right) \hfill \\ \Delta \dot{b}_{2} = - e_{3} - p_{1} \cdot sign\left( {\Delta b_{2} } \right)\left| {\Delta b_{2} } \right|^{{\mu_{1} }} - q_{1} \cdot \Delta b_{2} \hfill \\ \Delta \dot{c}_{2} = y_{3} e_{4} - p_{1} \cdot sign\left( {\Delta a_{1} } \right)\left| {\Delta c_{2} } \right|^{{\mu_{1} }} - q_{1} \cdot \left( {\Delta c_{2} } \right) \hfill \\ \Delta \dot{l}_{2} = - y_{4} e_{4} - p_{1} \cdot sign\left( {\Delta a_{1} } \right)\left| {\Delta l_{2} } \right|^{{\mu_{1} }} - q_{1} \cdot \left( {\Delta l_{2} } \right) \hfill \\ \end{gathered} \right. \hfill \\ \left\{ {\begin{array}{*{20}l} {\Delta \dot{d}_{1} = - e_{1} sign\left( {e_{1} } \right) - p_{1} \cdot sign\left( {\Delta d_{1} } \right)\left| {\Delta d_{1} } \right|^{{\mu_{1} }} - q_{1} \cdot \left( {\Delta d_{1} } \right)} \\ {\Delta \dot{d}_{2} = - e_{2} sign\left( {e_{2} } \right) - p_{1} \cdot sign\left( {\Delta d_{2} } \right)\left| {\Delta d_{2} } \right|^{{\mu_{1} }} - q_{1} \cdot \left( {\Delta d_{2} } \right)} \\ {\Delta \dot{d}_{3} = - e_{3} sign\left( {e_{3} } \right) - p_{1} \cdot sign\left( {\Delta d_{3} } \right)\left| {\Delta d_{3} } \right|^{{\mu_{1} }} - q_{1} \cdot \left( {\Delta d_{3} } \right)} \\ {\Delta \dot{d}_{4} = - e_{4} sign\left( {e_{4} } \right) - p_{1} \cdot sign\left( {\Delta d_{4} } \right)\left| {\Delta d_{4} } \right|^{{\mu_{1} }} - q_{1} \cdot \left( {\Delta d_{4} } \right)} \\ \end{array} } \right. \hfill \\ \end{gathered}$$

From Eq. (14), the controller can be designed as follows

35 $$\left\{ \begin{gathered} u_{1} = y_{2} + y_{3} + \hat{a}_{1} (x_{2} - x_{1} ) - \hat{d}_{1} sign\left( {e_{1} } \right) - p_{1} \cdot sign\left( {e_{1} } \right)\left| {e_{1} } \right|^{{\mu_{1} }} - q_{1} e_{1} + \hat{d}_{2} sign\left( {e_{2} } \right) + p_{1} \cdot sign\left( {e_{2} } \right)\left| {e_{2} } \right|^{{\mu_{1} }} + q_{1} e_{2} \hfill \\ u_{2} = - y_{1} - y_{4} - \hat{a}_{2} y_{2} + \hat{b}_{1} x_{1} + \hat{c}_{1} x_{2} - x_{1} x_{3} - \hat{d}_{2} sign\left( {e_{2} } \right) - p_{1} \cdot sign\left( {e_{2} } \right)\left| {e_{2} } \right|^{{\mu_{1} }} - q_{1} e_{2} \hfill \\ u_{3} = - y_{1} y_{3} - \hat{b}_{2} + \hat{b}_{1} x_{1} + \hat{c}_{1} x_{2} - x_{1} x{}_{3} + x_{1}^{2} - \hat{l}_{1} x_{3} - \hat{d}_{3} sign\left( {e_{3} } \right) - p_{1} \cdot sign\left( {e_{3} } \right)\left| {e_{3} } \right|^{{\mu_{1} }} - q_{1} e_{3} \hfill \\ u_{4} = \hat{c}_{2} y_{3} - \hat{l}_{2} y_{4} + \hat{a}_{1} (x_{2} - x_{1} ) + x_{1}^{2} - \hat{l}_{1} x_{3} - \hat{d}_{4} sign\left( {e_{2} } \right) - p_{1} \cdot sign\left( {e_{4} } \right)\left| {e_{4} } \right|^{{\mu_{1} }} - q_{1} le_{4} \hfill \\ \end{gathered} \right.$$

In the controller (35), the error dynamical system is as follows

36 $$\left\{ \begin{gathered} \dot{e}_{1} = - \Delta a_{1} \left( {x_{2} - x_{1} } \right) + \Delta a_{2} y_{2} - \Delta b_{1} x_{1} - \Delta c_{1} x_{2} - \tilde{d}_{1} sign\left( {e_{1} } \right) - p_{1} \cdot sign\left( {e_{1} } \right)\left| {e_{1} } \right|^{{\mu_{1} }} - q_{1} e_{1} \hfill \\ \dot{e}_{2} = \Delta a_{2} y_{2} - \Delta b_{1} x_{1} - \Delta c_{1} x_{2} - \tilde{d}_{2} sign\left( {e_{2} } \right) - p_{1} \cdot sign\left( {e_{2} } \right)\left| {e_{2} } \right|^{{\mu_{1} }} - q_{1} e_{2} \hfill \\ \dot{e}_{3} = \Delta b_{2} - \Delta b_{1} x_{1} - \Delta c_{1} x_{2} + \Delta l_{1} x_{3} - \tilde{d}_{3} sign\left( {e_{3} } \right) - p_{1} \cdot sign\left( {e_{3} } \right)\left| {e_{3} } \right|^{{\mu_{1} }} - q_{1} e_{3} \hfill \\ \dot{e}_{4} = - \Delta c_{2} y_{3} + \Delta l_{2} y_{4} - \Delta a_{1} \left( {x_{2} - x_{1} } \right) + \Delta l_{1} x_{3} - \tilde{d}_{4} sign\left( {e_{2} } \right) - p_{1} \cdot sign\left( {e_{4} } \right)\left| {e_{4} } \right|^{{\mu_{1} }} - q_{1} e_{4} \hfill \\ \end{gathered} \right.$$

**Figure 1:**
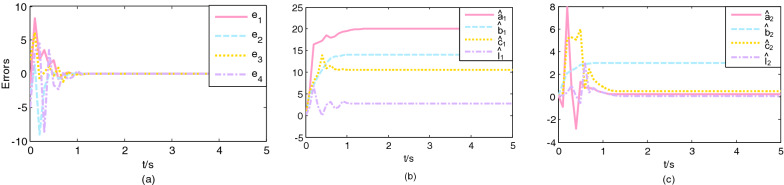
Trajectories: (**a**) errors; (**b**) the unknown parameter estimates of the Cai system; (**c**) the unknown parameter estimates of the Rossler system. $\left( {p_{1} = q_{1} = 2} \right)$.

**Figure 2:**
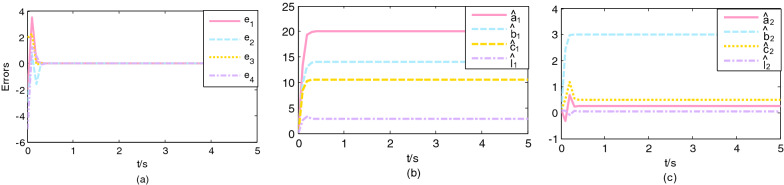
Trajectories: (**a**) errors; (**b**) the unknown parameter estimates of the Cai system; (**c**) the unknown parameter estimates of the Rossler system. $\left( {p_{1} = q_{1} = 8} \right)$.

The initial values are chosen $\left( {x_{1} \left( 0 \right),x_{2} \left( 0 \right),x_{3} \left( 0 \right)} \right) = \left( {4, - 3,4} \right),\left( {y_{1} \left( 0 \right),y_{2} \left( 0 \right),y_{3} \left( 0 \right),y_{4} \left( 0 \right)} \right) = \left( {5, - 6,3,3} \right)$, and the initial value of parameter estimations are $\left( {\hat{a}_{1} \left( 0 \right),\hat{b}_{1} \left( 0 \right),\hat{c}_{1} \left( 0 \right),\hat{l}_{1} \left( 0 \right)} \right) = \left( {0.1,0.1,0.1} \right)$, $\left( {\hat{a}_{2} \left( 0 \right),\hat{b}_{2} \left( 0 \right),\hat{c}_{2} \left( 0 \right),\hat{l}_{2} \left( 0 \right)} \right) = \left( {0.1,0.1,0.1,0.1} \right)$ and $\left( {\hat{d}_{1} \left( 0 \right),\hat{d}_{2} \left( 0 \right),\hat{d}_{3} \left( 0 \right),\hat{d}_{4} \left( 0 \right)} \right) = \left( {0.1,0.1,0.1,0.1} \right)$, two sets of gain constants are selected $p_{1} = 2,q_{1} = 2$ and $p_{1} = 8,q_{1} = 8$, $\mu_{1}$ is a constant of 0.5. The external disturbance function are selected ${\varvec{h}}\left( t \right) = \left( {0.01\sin \left( {10t} \right), - 0.02\cos \left( {10t} \right),0.05\sin \left( {10t} \right)} \right)^{T}$ and ${\varvec{H}}\left( t \right) = \left( {0.01\sin \left( {10t} \right), - 0.02\cos \left( {10t} \right),0.01\cos \left( {10t} \right),0.02\sin \left( {10t} \right)} \right)^{T}$. Figure 1a shows that the synchronization error of the error system with the controller gradually tends to zero in $T_{1} = 1.2809\;{\text{s}}$, and Fig. 2a shows that the synchronization error of the error system with the controller gradually tends to zero in $T_{1} = 0.3202\;{\text{s}}$. The variation of parameter estimates of the driving system and response system with time is shown in Figs. 1b, c and 2b, c, it can be seen that the parameter estimates also converge to the value in a finite time.

As shown in Figs. 1 and 2, if the control gain constant is large, the convergence speed of synchronization will be faster, that is, the time for the system to realize synchronization is shorter. The value of the control gain constant only affects the synchronization speed. The following example can take any set of experimental values in consideration of space.

### Remark 3.

In literature^28^, by adding dimensions to the drive system (26), the synchronization error form $e_{i} = y_{i} - x_{i - 1} \left( {i = 1,2,3,4} \right)$ of cross subtraction is constructed, and the appropriate controller and parameter adaptive laws are designed so that the drive system (30) and the response system (31) with unknown parameters can be synchronized in $4D$. By using Theorem 1 and selecting $$\Theta { = }\left[ {\begin{array}{*{20}l} 1 & 0 & 0 & 0 \\ 0 & 1 & 0 & 0 \\ 0 & 0 & 1 & 0 \\ 0 & 0 & 0 & 1 \\ \end{array} } \right],\Phi { = }\left[ {\begin{array}{*{20}l} 0 & 0 & 0 \\ 1 & 0 & 0 \\ 0 & 1 & 0 \\ 0 & 0 & 1 \\ \end{array} } \right]$$, $\rho = {\mathbf{0}},\rho^{\prime} = {\mathbf{0}}$, The external disturbance function are selected ${\varvec{h}}\left( t \right) = 0,{\varvec{H}}\left( t \right) = 0$, the controller and parameter adaptive laws designed in this paper are the same as that in literature^28^, that is, the $\Theta { - }\Phi$ synchronization in this paper can be transformed into the synchronization scheme in literature^28^. In literature^29^, the external disturbance function are selected ${\varvec{h}}\left( t \right) = 0,{\varvec{H}}\left( t \right) = 0$ , the controller and parameter adaptive laws designed in this paper are the same as that in literature^29^. Compared with the conclusion given in literature^28^, the constant gain matrices and gain function are added to the designed parameter adaptive laws, making the Theorem 1 and Corollary 1 given in this paper more universal. Compared with the conclusion given in literature^29^, we have added the disturbance function in the design of the control plane, which makes our conclusion more practical. More importantly, compared with the previous literatures, the time $T$ when the system reaches synchronization is also given.

### Example 2.

The financial system is described as follows:

37 $$\left\{ \begin{gathered} \dot{x}_{1} = x_{3} + \left( {x_{2} - r_{1} } \right)x_{1} \hfill \\ \dot{x}_{2} = 1 - r_{2} x_{2} - x_{1}^{2} \hfill \\ \dot{x}_{3} = - x_{1} - r_{3} x_{3} \hfill \\ \end{gathered} \right.$$

and the Chen-Lee systemis described as follows:

38 $$\left\{ \begin{gathered} \dot{y}_{1} = \omega_{1} y_{1} - y_{2} y_{3} + u_{1} \hfill \\ \dot{y}_{2} = \omega_{2} y_{2} + y_{1} y_{3} + u_{2} \hfill \\ \dot{y}_{3} = \omega_{3} y_{3} + 0.2y_{4} + \frac{1}{3}y_{1} y_{2} + u_{3} \hfill \\ \dot{y}_{4} = 2.2y_{1} + 0.05y_{4} + 0.5y_{2} y_{3} + u_{4} \hfill \\ \end{gathered} \right.$$

where the parameters are selected as $r_{1} = 0.8,r_{2} = 0.2,r_{3} = 1.9$ and $\omega_{1} = 5,\omega_{2} = - 10,\omega_{3} = - 3.8$, the system (37) and (38) are chaotic and hyperchaotic, respectively^26,27^. Rewrite the system Eqs. (37) and (38) with external disturbances and uncertain parameters into the following forms to synchronize them in four-dimensions.

39 $$\dot{\user2{x}} = A_{{\varvec{\alpha}}} {\varvec{x}} + F\left( {\varvec{x}} \right) + {\varvec{h}}\left( t \right)$$

where $$A_{{\varvec{\alpha}}} = \left[ {\begin{array}{*{20}l} {r_{1} } & 0 & 0 \\ 0 & {r_{2} } & 0 \\ 0 & 0 & {r_{3} } \\ \end{array} } \right]$$, $$F\left( {\varvec{x}} \right) = \left[ {\begin{array}{*{20}l} {x_{3} + x_{1} x_{2} } \\ {1 - x_{1}^{2} } \\ { - x_{1} } \\ \end{array} } \right]$$.

40 $$\dot{\user2{y}}{ = }B_{{\varvec{\beta}}} {\varvec{y}} + G\left( {\varvec{y}} \right) +{\varvec{H}}\left( t \right) + {\varvec{u}}$$

where $$B_{{\varvec{\beta}}} = \left[ {\begin{array}{*{20}l} {\omega_{1} } & 0 & 0 & 0 \\ 0 & {\omega_{2} } & 0 & 0 \\ 0 & 0 & {\omega_{3} } & 0 \\ 0 & 0 & 0 & 0 \\ \end{array} } \right]$$, $$G\left( {\varvec{y}} \right) = \left[ {\begin{array}{*{20}l} { - y_{2} y_{3} } \\ {y_{1} y_{3} } \\ {0.2y_{4} + \frac{1}{3}y_{1} y_{2} } \\ {2.2y_{1} + 0.05y_{4} + 0.5y_{2} y_{3} } \\ \end{array} } \right]$$.

We choose $$\Theta { = }\left[ {\begin{array}{*{20}l} 1 & 0 & 0 & 0 \\ 0 & 1 & 0 & 0 \\ 0 & 0 & 1 & 0 \\ 1 & 0 & 0 & 1 \\ \end{array} } \right],\Phi { = }\left[ {\begin{array}{*{20}l} 1 & 0 & 0 \\ 0 & 1 & 0 \\ 0 & 0 & 1 \\ 1 & 1 & 0 \\ \end{array} } \right]$$. From Eq. (25), the adaptive laws of parameters can be designed as follows:

41 $$\begin{gathered} \left\{ \begin{gathered} \Delta \dot{r}_{1} = e_{1}^{2} { + }e_{1} e_{4} - p_{2} \cdot sign\left( {\Delta r_{1} } \right)\left| {\Delta r_{1} } \right|^{{\mu_{2} }} - q_{2} \cdot \left( {\Delta r_{1} } \right) \hfill \\ \Delta \dot{r}_{2} = e_{2}^{2} { + }e_{2} e_{4} - p_{2} \cdot sign\left( {\Delta r_{2} } \right)\left| {\Delta r_{2} } \right|^{{\mu_{2} }} - q_{2} \cdot \left( {\Delta r_{2} } \right) \hfill \\ \Delta \dot{r}_{3} = e_{3}^{2} - p_{2} \cdot sign\left( {\Delta r_{3} } \right)\left| {\Delta r_{3} } \right|^{{\mu_{2} }} - q_{2} \cdot \left( {\Delta r_{3} } \right) \hfill \\ \end{gathered} \right. \hfill \\ \left\{ \begin{gathered} \Delta \dot{\omega }_{1} = - e_{1}^{2} - e_{1} e_{4} - p_{2} \cdot sign\left( {\Delta \omega_{1} } \right)\left| {\Delta \omega_{1} } \right|^{{\mu_{2} }} - q_{2} \cdot \left( {\Delta \omega_{1} } \right) \hfill \\ \Delta \dot{\omega }_{2} = - e_{2}^{2} - p_{2} \cdot sign\left( {\Delta \omega_{2} } \right)\left| {\Delta \omega_{2} } \right|^{{\mu_{2} }} - q_{2} \cdot \left( {\Delta \omega_{2} } \right) \hfill \\ \Delta \dot{\omega }_{3} = - e_{3}^{2} - p_{2} \cdot sign\left( {\Delta \omega_{3} } \right)\left| {\Delta \omega_{3} } \right|^{{\mu_{2} }} - q_{2} \cdot \left( {\Delta \omega_{3} } \right) \hfill \\ \end{gathered} \right. \hfill \\ \left\{ {\begin{array}{*{20}l} {\Delta \dot{d}_{1} = - e_{1} sign\left( {e_{1} } \right) - p_{2} \cdot sign\left( {\Delta d_{1} } \right)\left| {\Delta d_{1} } \right|^{{\mu_{2} }} - q_{2} \cdot \left( {\Delta d_{1} } \right)} \\ {\Delta \dot{d}_{2} = - e_{2} sign\left( {e_{2} } \right) - p_{2} \cdot sign\left( {\Delta d_{2} } \right)\left| {\Delta d_{2} } \right|^{{\mu_{2} }} - q_{2} \cdot \left( {d_{2} } \right)} \\ {\Delta \dot{d}_{3} = - e_{3} sign\left( {e_{3} } \right) - p_{2} \cdot sign\left( {\Delta d_{3} } \right)\left| {\Delta d_{3} } \right|^{{\mu_{2} }} - q_{2} \cdot \left( {\Delta d_{3} } \right)} \\ {\Delta \dot{d}_{4} = - e_{4} sign\left( {e_{4} } \right) - p_{2} \cdot sign\left( {\Delta d_{4} } \right)\left| {\Delta d_{4} } \right|^{{\mu_{2} }} - q_{2} \cdot \left( {\Delta d_{4} } \right)} \\ \end{array} } \right. \hfill \\ \end{gathered}$$

From Eq. (24), the controller can be designed as follows:

42 $$\left\{ \begin{gathered} u_{1} = y_{2} y_{3} + x_{2} x_{1} + x_{3} - \omega_{1} x_{1} - r_{1} y_{1} - \hat{\omega }_{1} e_{1} + \hat{r}_{1} e_{1} - \hat{d}_{1} sign\left( {e_{1} } \right) - p_{2} \cdot sign\left( {e_{1} } \right)\left| {e_{1} } \right|^{{\mu_{2} }} - q_{2} \cdot \left( {e_{1} } \right) \hfill \\ u_{2} = - y_{1} y_{3} + 1 - x_{1}^{2} - \omega_{2} x_{2} - r_{2} y_{2} - \hat{\omega }_{2} e_{2} + \hat{r}_{2} e_{2} - \hat{d}_{2} sign\left( {e_{2} } \right) - p_{2} \cdot sign\left( {e_{2} } \right)\left| {e_{2} } \right|^{{\mu_{2} }} - q_{2} \cdot \left( {e_{2} } \right) \hfill \\ u_{3} = - 0.2y_{4} - \frac{1}{3}y_{1} y_{2} - x_{1} - \omega_{3} x_{3} - r_{3} y_{3} - \hat{\omega }_{3} e_{3} + \hat{r}_{3} e_{3} - \hat{d}_{3} sign\left( {e_{3} } \right) - p_{2} \cdot sign\left( {e_{3} } \right)\left| {e_{3} } \right|^{{\mu_{2} }} - q_{2} \cdot \left( {e_{3} } \right) \hfill \\ u_{4} = - 2.2y_{1} - 0.05y_{4} - 0.5y_{2} y_{3} + 1 - x_{1}^{2} - r_{2} y_{2} + \hat{r}_{2} e_{2} - \hat{d}_{4} sign\left( {e_{4} } \right) - p_{2} \cdot sign\left( {e_{4} } \right)\left| {e_{4} } \right|^{{\mu_{2} }} - q_{2} \cdot \left( {e_{4} } \right) \hfill \\ \end{gathered} \right.$$

In the controller (42), the error dynamical system is as follows:

43 $$\left\{ \begin{gathered} \dot{e}_{1} = \left( {\Delta \omega_{1} - \Delta r_{1} } \right)e_{1} - \hat{d}_{1} sign\left( {e_{1} } \right) - p_{2} \cdot sign\left( {e_{1} } \right)\left| {e_{1} } \right|^{{\mu_{2} }} - q_{2} \cdot \left( {e_{1} } \right) \hfill \\ \dot{e}_{2} = \left( {\Delta \omega_{2} - \Delta r_{2} } \right)e_{2} - \hat{d}_{2} sign\left( {e_{2} } \right) - p_{2} \cdot sign\left( {e_{2} } \right)\left| {e_{2} } \right|^{{\mu_{2} }} - q_{2} \cdot \left( {e_{2} } \right) \hfill \\ \dot{e}_{3} = \left( {\Delta \omega_{3} - \Delta r_{3} } \right)e_{3} - \hat{d}_{3} sign\left( {e_{3} } \right) - p_{2} \cdot sign\left( {e_{3} } \right)\left| {e_{3} } \right|^{{\mu_{2} }} - q_{2} \cdot \left( {e_{3} } \right) \hfill \\ \dot{e}_{4} = \left( {\Delta \omega_{1} - \Delta r_{1} } \right)e_{1} - \Delta r_{2} e_{2} - \hat{d}_{4} sign\left( {e_{4} } \right) - p_{2} \cdot sign\left( {e_{4} } \right)\left| {e_{4} } \right|^{{\mu_{2} }} - q_{2} \cdot \left( {e_{4} } \right) \hfill \\ \end{gathered} \right.$$

**Figure 3:**
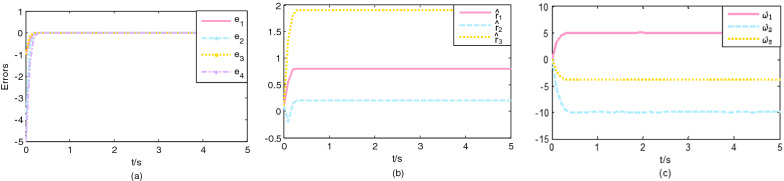
Trajectories: (**a**) errors; (**b**) the unknown parameter estimates of the financial system; (**c**) the unknown parameter estimates of the Chen-Lee system. $\left( {p_{{2}} = q_{{2}} = {6}} \right)$.

The initial values are chosen $$\left( {x_{1} \left( 0 \right),x_{2} \left( 0 \right),x_{3} \left( 0 \right)} \right) = \left( {2,6,4} \right)$$ and $$\left( {y_{1} \left( 0 \right),y_{2} \left( 0 \right),y_{3} \left( 0 \right),y_{4} \left( 0 \right)} \right) = \left( {1,2,3,2} \right)$$, the initial value of parameter estimations are $$\left( {\hat{r}_{1} \left( {0} \right){,}\hat{r}_{2} \left( {0} \right){,}\hat{r}_{3} \left( 0 \right)} \right) = \left( {0.1,0.1,0.1} \right)$$, $$\left( {\hat{\omega }_{1} \left( 0 \right),\hat{\omega }_{2} \left( 0 \right),\hat{\omega }_{3} \left( 0 \right)} \right) = \left( {0.1,0.1,0.1} \right)$$ and $$\left( {\hat{d}_{1} \left( 0 \right),\hat{d}_{2} \left( 0 \right),\hat{d}_{3} \left( 0 \right),\hat{d}_{4} \left( 0 \right)} \right) = \left( {0.1,0.1,0.1,0.1} \right)$$, gain constants are selected $$p_{2} = 6,q_{2} = 6$$, $$\mu_{2}$$ is a constant of 0.5. The external disturbance function are chosen as $${\varvec{h}}\left( t \right) = \left( {0.05\sin \left( {5t} \right), - 0.01\sin \left( {5t} \right),0.02\cos \left( {5t} \right)} \right)^{T}$$$${\varvec{H}}\left( t \right) = \left( {0.01\cos \left( {5t} \right), - 0.02\cos \left( {5t} \right), - 0.01\cos \left( {5t} \right),0.02\sin \left( {5t} \right)} \right)^{T}$$. Figure 3a shows that the synchronization error of the error system with the controller gradually tends to zero in $$T_{2} = 0.3988s$$. The variation of parameter estimates of the driving system and response system with time is shown in Fig. 3b, c, it can be seen that the parameter estimates also converge to the value in a finite time.

## Conclusions

This paper realized finite-time synchronization of different dimensional chaotic systems with external disturbances and uncertain parameters. Based on the characteristics of chaotic systems, several synchronization schemes are given using the scaling matrices. In all, numerical experiments have been employed to validate the proposed methods. Although some conclusions of finite-time synchronization of chaotic systems are obtained in this paper, the obtained control scheme is designed based on the adaptive control method. Our future work will continue to design more novel and convenient synchronization schemes according to the characteristics of chaotic systems and the method of sliding mode control.

## Data Availability

The main results of our work are proved in detail, which can be seen in the context. No data were used to support this study.

## References

[1] Allegro KT, Sauer TD (1997). Yorke: Chaos: An Introduction to Dynamical Systems.

[2] Pecora LM, Carroll TL (1990). Synchronization in chaotic systems. Phys. Rev. Lett..

[3] Manjit K, Vijay K (2018). Adaptive differential evolution-based lorenz chaotic system for image encryption. Arab. J. Sci. Eng..

[4] Saleh M, Sundarapandian V, Aceng S (2019). A novel chaotic system with boomerang-shaped equilibrium, its circuit implementation and application to sound encryption. Iran. J. Sci. Technol. Trans. Electr. Eng..

[5] Talatahari S, Azizi M (2020). Optimization of constrained mathematical and engineering design problems using chaos game optimization. Comput. Ind. Eng..

[6] Lee K, Raymond ST (2020). Chaotic type-2 transient-fuzzy deep neuro-oscillatory network (CT2TFDNN) for worldwide financial prediction. IEEE Trans. Fuzzy Syst..

[7] Haimo VT (1986). Finite time controller. SIAM J. Control Optim..

[8] Shen Y, Huang Y (2012). Global finite-time stabilisation for a class of nonlinear systems. Int. J. Syst. Sci..

[9] Wang X, Mia P (2020). Finite-time function projective synchronization in complex multi-links networks and application to Chua’s circuit. Int. J. Control Autom. Syst..

[10] Sangpet T, Kuntanapreeda S (2020). Finite-time synchronization of hyperchaotic systems based on feedback passivation. Chaos Solit. Fract..

[11] Haris M (2021). A Nonlinear adaptive controller for the synchronization of unknown identical chaotic systems. Arab. J. Sci. Eng..

[12] Pan WQ, Li TZ (2021). Finite-time synchronization of fractional-order chaotic systems with different structures under stochastic disturbances. J. Comput. Commun..

[13] Lin ML, Yuan ZZ, Cai JP (2019). Finite-time synchronization between two different chaotic systems with uncertainties. J. Fujian Univ. Technol..

[14] Stefanovska A, Haken H, McClintock PVE (2000). Reversible transitions between synchronization states of the cardiorespiratory system. Phys. Rev. Lett..

[15] Alvarez G, Hernández L, Muñoz J (2005). Security analysis of communication system based on the synchronization of different order chaotic systems. Phys. Lett. A.

[16] Samuel BW, McClintock PVE (2006). Adaptive synchronization between chaotic dynamical systems of different order. Phys. Lett. A.

[17] Ouannas A, Al-sawalha MM (2016). Synchronization between different dimensional chaotic systems using two scaling matrices. Optik.

[18] Cai N, Li WQ, Jing YW (2011). Finite-time generalized synchronization of chaotic systems with different order. Nonlinear Dyn..

[19] Zhao JK, Wu Y, Wang YY (2013). Generalized finite-time synchronization between coupled chaotic systems of different orders with unknown parameters. Nonlinear Dyn..

[20] Guo XZ, Wen GG, Peng ZX (2020). Global fixed-time synchronization of chaotic systems with different dimensions. J. Franklin Inst..

[21] Ahmad I, Shafiq M, Saaban AB (2016). Robust finite-time global synchronization of chaotic systems with different orders. Optik.

[22] Dong N (2009). Adaptive Control.

[23] Khalil HK (2002). Nonlinear Systems.

[24] Cai GL, Tan ZM, Zhou WH (2007). Dynamical analysis of a new chaotic system and its chaotic control. Acta Phys. Sin..

[25] Rossler OE (1976). An equation for continuous chaos. Phys. Lett. A.

[26] Ma JH, Chen YS (2001). Study for the bifurcation topological structure and the global complicated character of a kind of non-linear finance system. Appl. Math. Mech..

[27] Chen YW (2009). Finite time synchronization of hyperchaotic Chen-Lee system. J. Zhangzhou Normal Univ. Nat. Sci..

[28] Gao J, Zhang XH (2013). Synchronization of chaotic systems with different dimensions based on adaptive control. Comput. Eng. Sci..

[29] Zheng JM, Li J (2021). Synchronization of a class of chaotic systems with different dimensions. Complexity.

